# The Role of the Intestinal Flora and Its Derivatives in Neurocognitive Disorders: A Narrative Review from Surgical Perspective

**DOI:** 10.1007/s12035-024-04322-1

**Published:** 2024-07-10

**Authors:** Jian Huang, Tian-Shou Qin, Yun Bo, Yu-Jin Li, Rong-Sheng Liu, Yang Yu, Xiao-Dong Li, Jin-Can He, Ai-Xin Ma, Da-Peng Tao, Wen-Jun Ren, Jun Peng

**Affiliations:** 1 The First People’s Hospital of Yunnan Province, The Affiliated Hospital of Kunming University of Science and Technology, Kunming, 650032 People’s Republic of China; 2 Department of Thoracic Surgery, The First People’s Hospital of Yunnan Province, The Affiliated Hospital of Kunming University of Science and Technology, Kunming, 650032 China; 3 Department of Cardiovascular Surgery, The First People’s Hospital of Yunnan Province, The Affiliated Hospital of Kunming University of Science and Technology, Kunming, 650032 Yunnan China; 4 Department of Anesthesiology, The First People’s Hospital of Yunnan Province, The Affiliated Hospital of Kunming University of Science and Technology, Kunming, 650032 China; 5 School of Information Science and Engineering, Yunnan University, Kunming, 650504 China

**Keywords:** Perioperative neurocognitive dysfunction (PND), Postoperative cognitive complications, Neuropsychological tests, Cognitive dysfunction, Gut microbiota (GM), Extracellular vesicles (EVs), Gut–brain axis, Surgery

## Abstract

Perioperative neurocognitive dysfunction is a significant concern for population health, impacting postoperative recovery and increasing the financial burden on patients. With an increasing number of surgical procedures being performed, the prevention and management of perioperative neurocognitive dysfunction have garnered significant attention. While factors such as age, lifestyle, genetics, and education are known to influence the development of cognitive dysfunction, recent research has highlighted the role of the gut microbiota in neurological health. An increased abundance of pro-inflammatory gut microbiota can trigger and worsen neuroinflammation, neuronal cell damage, and impaired cellular autophagy. Moreover, the inflammation-promoting gut microbiota can disrupt immune function, impair neuroautophagy, and affect the production and circulation of extracellular vesicles and neurotransmitters. These factors collectively play a role in the onset and advancement of cognitive impairment. This narrative review delves into the molecular mechanisms through which gut microbiota and their derivatives contribute to cognitive impairment, focusing on the impact of anesthesia surgery, changes in gut microbial populations, and perioperative cognitive impairment associations. The study suggests that alterations in the abundance of various bacterial species and their metabolites pre- and post-surgery may be linked to postoperative cognitive impairment. Furthermore, the potential of probiotics or prebiotics in addressing cognitive impairment is discussed, offering a promising avenue for investigating the treatment of perioperative neurocognitive disorders.

## Introduction

Perioperative neurocognitive dysfunction (PND), which encompasses preoperative cognitive dysfunction, postoperative delirium, delayed neurocognitive recovery, and postoperative neurocognitive disorder, commonly affects elderly patients [1, 2]. Age is a major risk factor for PND, with cognitive impairment occurring more frequently in elderly patients than in younger patients undergoing surgery [3]. Patients who undergo cardiovascular and orthopedic surgeries have a greater incidence of postoperative cognitive disorders, with approximately 40% of cardiovascular surgery patients experiencing PND [4], while approximately 23.8% of noncardiovascular surgery patients experience postoperative cognitive disorders [5]. Factors such as education level, type of anesthesia, and postoperative pain also contribute to the risk of PND [6–10]. The pathogenesis of PND remains unclear, with current theories focusing on factors such as intraoperative and postoperative microembolism, neuroinflammation, oxidative stress, neuronal demodulation, synaptic structural plasticity damage, abnormal Tau protein modification, and neurotrophic factor deprivation [11, 12]. Interestingly, the study by Glumac et al. [13] has shown that preoperative administration of dexamethasone before cardiac surgery significantly decreases the occurrence of postoperative neurocognitive deficits in patients. The suggested mechanism for this protective effect is by reducing neuroinflammatory responses and enhancing tolerance to perioperative stress. This evidence highlights the crucial role of neuroinflammation in the development of cognitive impairment. PND poses a serious threat to the health and quality of life of elderly individuals, leading to prolonged hospitalization, increased patient mortality, and a heightened societal burden. Moreover, specific treatments for PND are lacking, and drug treatments like dexamethasone have shown effectiveness in improving postoperative cognitive impairment, alongside non-drug interventions such as sleep management, cognitive training, music therapy, close nursing care, monitoring, and traditional Chinese medicine available [14–16]. Therefore, further research into the pathogenesis of PND is crucial for developing effective prevention and treatment strategies.

Microbiome research has gained significant attention in recent years, highlighting the intricate connection between microbial composition and human health. This relationship is characterized by direct microbial-host interactions, the modulation of immune and inflammatory responses, the influence of microorganisms and their metabolites on cellular signaling pathways, and potential genotoxic effects [17]. Studies have indicated strong correlations between the composition of the gut microbiota (GM) and both the nervous system [18] and psychosomatic health [19, 20]. The GM plays a crucial role in fermenting dietary fiber to produce short-chain fatty acids (SCFAs), which not only provide energy to the intestinal mucosa but also promote intestinal peristalsis for waste elimination [21]. Disruption of the GM can compromise the intestinal mucosal barrier, leading to the translocation of toxins and metabolites into the bloodstream and triggering inflammatory responses that may impact the central nervous system [22]. Recent research has demonstrated that surgical and anesthetic procedures can alter the GM composition [23–25], hypothetically contributing to the development of PND. Thus, analyzing perioperative changes in the intestinal flora and exploring interventions based on these alterations could offer a promising approach to preventing and managing neurocognitive disorders. This narrative review aimed to explore the relationship between the gut microbiome, its metabolic products, and neurocognitive impairment. The focus is on understanding how changes in the gut microbial ecosystem contribute to cognitive deficits. Specifically, we highlight the impact of perioperative gut microbiome dysbiosis on neurocognitive complications. Based on current research, we suggest that manipulating the gut microbiome could be a potential treatment for perioperative neurocognitive disorders.

## Methods

A thorough literature search was conducted across PubMed, Web of Science, and CNKI databases for studies up to May 2024. The search utilized key terms related to postoperative cognitive dysfunction, perioperative neurocognitive disorders, cognitive impairment, and the gut microbiome. Both human and animal studies were considered, with initial screening done by J H and TS Q and final selections made by Y B and WJ R. Reference lists of relevant studies were also reviewed for additional publications. Due to the diverse nature of study populations, species, and outcome measures, a narrative review approach was chosen over a systematic one. Inclusion criteria focused on studies exploring the link between neurocognitive impairment and the gut microbiome, with accessibility to full-text articles being a key factor. Exclusion criteria encompassed non-English or non-Chinese publications, correspondence, conference proceedings, and retracted studies.

## Results

### Characterization of the Gut Microbiota in Patients with Cognition Disorders

Numerous studies have shown notable differences in GM composition between individuals with and without cognitive impairment. Kaiyrlykyzy et al. [26] reported that patients with Alzheimer’s disease (AD) had greater abundances of *Acidobacteriota*, *Verrucomicrobiota*, *Synergistota*, and *Planctomycetota*, among others, than healthy individuals. Additionally, the *Prevotella*, *Alloprevotella*, and *Ruminococcus* genera were more abundant, while the abundances of *Bifidobacterium*, *Clostridia bacterium*, and *Lactiplantibacillus* were significantly reduced. Bairamian et al. [27] reported that patients with AD exhibited increases in proinflammatory flora (e.g., *Bacteroidetes*) and decreases in anti-inflammatory flora (e.g., the *Firmicutes* phylum), revealing potential variations based on geography and population. Zhang and other scholars [28] have conducted preclinical experiments similar to those in previous studies. They discovered that the intestinal microbiota of aged mice with cognitive dysfunction postsurgery differed significantly from that of normal mice. Specifically, certain genera of *Bacteroidetes* showed notable increases, while bacterial genera such as *Lachnospiraceae bacterium A2* and *Blautia* decreased significantly. Metabolomic analysis revealed decreasing trends in metabolites such as thiamine and long-chain unsaturated fatty acids. It is worth noting that the declining genera are responsible for producing metabolites such as SCFAs, which are beneficial for host health. This suggests that the metabolites of the gut flora play a crucial role in maintaining overall health. A recent study from Korea [29] revealed that patients with dementia who underwent fecal microbial transplants exhibited increased levels of *Bacteroides*, *Alistipes*, and *Odoribacter*, with a decrease in *Enterococcaceae* abundance. Additionally, changes in the expression levels of lipid metabolism genes were observed, potentially contributing to cognitive impairment. These contrasting results underscore the intricate interactions between the host and microbial communities and their combined effects on disease development. The human microbiota exhibits functional redundancy, meaning that changes in a specific strain may not always lead to drastic alterations in physiological function. Moreover, various strains of bacteria within the same genus can interact with the human body in diverse ways. Thus, investigating the impact of the microbiota on human genes and metabolism appears to be more crucial than solely focusing on microbiota changes. Additionally, variations in host genetics and metabolism can influence how the microbiota responds when similar diseases affect different individuals. Overall, patients with cognition disorders may have a GM that is functionally proinflammatory, characterized by an increase in inflammatory flora and a simultaneous decrease in anti-inflammatory flora (Table 1).

**Table 1:** Characterization of the gut microbiota in cognitively impaired individuals

Research teams	Species	Diseases	Findings	Reference
Yamashiro et al	Human	Alzheimer’s disease	*Anaerostipes*↓, *Roseburia*↓, *Lachnospiraceae UCG-004*↓, *Ruminococcaceae UCG-013*↓, *Eisenbergiella*↑	[91]
Laske et al	Human	Alzheimer’s disease	*Clostridium↑*, *Mediterranea↑*, *Erysipelatoclostridium↑*	[92]
Zhang et al	Mice	Postoperative cognitive dysfunction	*Bacteroidetes unclassified↑*, *Bacteroides acidifaciens↑*, *Rikenellaceae bacterium↑ *et al*.*, *Lachnospiraceae bacterium A2*↓, *Lachnospiraceae bacterium A4*↓, *Lachnospiraceae bacterium*↓ et al.	[28]
Pan et al	Mice	Postoperative cognitive dysfunction	Cognitive disorders in aged mice *p_Bacteroidetes*↑, *g_Akkermansia*↓	[74]
Kim et al	Human	Mild cognitive impairment	*Eubacterium nodatum group↑*, *Oribacterium*, *Rikenellaceae RC9 gut group↑*, *Bacteroides↑*, *Prevotella↓*, *Coprococcus↓*, *Akkermansia↓*, *Lachnospiraceae UCG-010↓*, *Prevotellaceae UCG-001↓*,* Clostridia UCG-014↓*	[93]
McLeod et al	Human	Mild cognitive impairment	*Str eptococcus↑*, *Ruminococcaceae UCG-002↑*, *Methanobrevibacter↑*, *Bifidobacterium↑*, *Dialister invisus↑*, *Parabacteroides distasonis↓*	[94]
Zhu et al	Human	Alzheimer’s disease	*Erysipelatoclostridiaceae↑*, *Erysipelotrichales↑*, *Saccharimonadales↑*, *Patescibacteria↑*,* Saccharimonadia↑*	[95]
Khedr et al	Human	Alzheimer’s disease	*Akkermansia↑*, *Enterobacteria↑*, *Bacteroidetes↑*, *Bacillus cereus↑*, *Prevotella↑*, *Clostridium cluster IV↑*, *Bifidobacterium spp↓*, *Firmicutes*,* Actinobacteria↓*	[96]
Pan et al	Human	Alzheimer’s disease	*Bacteroides salyersiae↓*, *Sphingobacterium multivorum↓*, *Leptotrichia buccalis↑*, *Staphylococcus intermedius↑*	[97]
Lian et al	Mice	Postoperative cognitive dysfunction	*Escherichia–Shigella↑*, *Fusobacterium↑*, *Bacteroidales_unclassified↓*,* Lachnospiraceae_UCG-001↓*	[98]

Characterization of the gut microbiota in cognitively impaired individuals: (1) “↑” represents an increase in the abundance of this type of bacteria in individuals with this disease among this group of study subjects. (2) “↓” represents a decrease in the abundance of this type of bacteria in individuals with this disease among this group of study subjects

### Gut Microorganisms and Their Metabolites Are Closely Related to Body Metabolism

The number of microorganisms in the human body is nearly equal to the number of cells in the body [30], with a significant portion residing in the gut microbiota estimated to be approximately 10^14^ [31]. GM and its metabolites play a crucial role in human health, impacting various aspects, such as growth, development, nutrition, immunity, and metabolism. Research by Bar et al. [32] indicated that a substantial percentage of plasma metabolites can be linked to the composition of the GM, suggesting a profound influence on substance metabolism in the bloodstream. Metabolites produced by microorganisms that travel from the intestines to the circulation can disrupt normal cellular metabolism and immune function. Additionally, studies by Schirmer et al. [33] revealed a notable association between the expression levels of certain cytokines, such as IL-1β, IL-6, and IFNγ, and specific GM populations, highlighting the close relationship between cytokine production and GM. Liu et al. [34] identified variations in the gut microbiota and serum metabolites among populations at varying altitudes. Their findings highlighted a negative correlation between amino acid metabolism, specifically L-glutamine and L-valine metabolism, and the abundance of *Bacteroidetes* and a positive correlation with the abundance of *Proteobacteria*. These findings suggest that GM composition plays a crucial role in an individual’s physiological and metabolic functions. Moreover, research by Richards et al. [35] showed that different intestinal flora can impact colonic epithelial cells, resulting in distinct patterns of gene expression. This indicates that various microorganisms may have differing effects on human gene regulation, with specific microbiota potentially influencing specific gene expression. For example, *Fusobacterium nucleatum* was found to modulate macrophages through the *TNFSF9/TRAF1* signaling pathways [36]. Consequently, alterations in gene expression levels induced by GM can result in variations in downstream protein expression and subsequent biological responses.

### Gut Mucosal Barrier Disruption due to Microecological Imbalance Increases Central Nervous System Inflammation

The close relationship between the GM and its metabolites and the central nervous system has led to the development of the concept known as the “gut–brain axis.” Chidambaram et al. [37] discussed how the microbiota can stimulate the production and release of abnormal neurotransmitters, impacting cellular metabolism in the central nervous system through direct interactions with nerves such as the enteric and vagus nerves. The resulting signals from this process reach the brain. Wang et al. [38] suggested that, beyond direct anatomical connections, mechanisms of the gut–brain communication may involve the neuroendocrine–hypothalamic–pituitary axis, the intestinal immune system, neurotransmitters, the intestinal mucosal barrier, and the blood‒brain barrier. Furthermore, microorganisms play roles in generating active metabolites such as SCFAs, valeric acid, dopamine, and extracellular vesicles (EVs) of intestinal microbial origin [39, 40]. These metabolites enter the bloodstream and impact the central nervous system, subsequently influencing host cognition and behavior.

#### Intestinal Mucosal Barrier and the Blood‒Brain Barrier

The intestinal mucosal barrier consists of several layers, comprising an aqueous membrane layer within the intestinal lumen, a glycocalyx and mucus layer, a tightly connected mucosal epithelial layer, a mucosal lamina propria, and immune proteins and cells [41]. Bacteriostatic peptides abound within the mucus layer [42]. This barrier plays a crucial role in screening microorganisms and their metabolites from entering the circulation [40]. It not only preserves the structural integrity of the intestinal tract and facilitates nutrient absorption but also impedes the access of harmful bacteria and their metabolites to the circulation. Research has indicated that the GM can modulate the expression of choroid plexus genes [43], resulting in the production of SCFAs that augment the expression of tight junction proteins such as ZO-1 and OCLN. These proteins assist in preserving the integrity of the blood‒brain barrier and facilitating the clearance of Aβ proteins by microglia, thus mitigating neuroinflammation [44, 45]. Disruption of the intestinal mucosal barrier leads to heightened permeability, enabling harmful flora and toxic metabolites to enter the circulation, thereby eliciting the body’s immune response [46, 47] and influencing the tight junctions between neuronal cells. This disruption indirectly affects the permeability of the blood‒brain barrier.

#### Immune Dysregulation Induced by the Intestinal Flora Accelerates Cognitive Impairment

Neuroinflammation has been identified as a key mechanism contributing to cognitive impairment, with a substantial body of clinical evidence highlighting the significant impact of immune dysfunction on cognitive decline. In their review article, Loh et al. [48] emphasized the role of microglial and astrocyte dysfunction in the progression of various neurodegenerative diseases, such as AD, Parkinson’s disease, amyotrophic lateral sclerosis, and Huntington’s disease. Guo et al. [49] further discussed the differential expression of immune-related genes, such as CD177 and S100A12, in patients with AD compared to those with mild cognitive dysfunction, highlighting their crucial roles in neutrophil activation. Additionally, Zang et al. [50] demonstrated that altered gene expression related to centrocyte activation and interferon synthesis can impact cognitive abilities, particularly in depressed patients. The significance of immune factors in cognitive disorders is evident, with recent research emphasizing the role of the gut flora in maintaining immune balance. Early colonization by gut microbes influences immune cell development [51], with long-lasting effects on host health [52]. In the central nervous system, microglia and astrocytes play vital roles as immune cells, and their dysfunction leads to neuroinflammation and negative effects on overall health. Microglia help reduce neuroinflammation by phagocytosing proteins such as Aβ, tau, and α-synuclein [48], while astrocytes are involved in neurotransmitter regulation, signaling, and inflammatory responses within the brain [53]. In animal experiments, it has been observed that dysregulation of the gut microecology can lead to the abnormal activation of microglia and a decrease in synaptic plasticity [54, 55]. This relationship indicates that gut microbes may impact the function of microglia and astrocytes and that the malfunction of these cells can worsen neuroinflammation and result in cognitive impairments.

#### Microecological Dysregulation Causes Neurotransmitter Abnormalities in the Somatic Circulation

Neurotransmitters play a crucial role in the neurological functioning of the body and are essential for normal physiological metabolism. Abnormalities in certain neurotransmitters can lead to declines in learning and memory, while some abnormalities can contribute to neuroinflammation [56]. Recent research has suggested a connection between the gut microbiota and neurotransmitter production and release. Lynch and Hsiao [57] investigated how the microbiota can activate inactive neurotransmitters and synthesize or degrade neurotransmitters through biotransformation. Konstanti and colleagues [58] demonstrated that *Akkermansia muciniphila* can produce glutamic acid decarboxylase, which helps in the synthesis of γ-aminobutyric acid, leading to the inhibition of neuroexcitation. Furthermore, microbiota-derived metabolites play a role in host substance metabolism. For instance, Palepu et al. [59] reported in a preclinical study that SCFAs produced by the intestinal flora from the breakdown of dietary fibers can regulate the metabolism of substances such as tryptophan and neurotransmitters. Imbalances in the intestinal microbiota can alter the production and release of neurotransmitters through changes in these derived metabolites. Wang et al. [60] reported that exposure to methylmercury led to notable alterations in the diversity and composition of the intestinal microbiota in mice, resulting in dysbiosis that influenced the release of neurotransmitters such as 5-HT and dopamine into the bloodstream. As a result, it is plausible that irregular metabolism triggered either directly or indirectly by gut microbes could play a role in the onset of cognitive dysfunction.

#### Dysbiosis of the Intestinal Microbiota Alters EVs Originating from the Microbiome

EVs of bacterial origin serve as a crucial medium for communication among different bacterial colonies and between colonies and hosts. These vesicles primarily consist of lipopolysaccharides, lipids, and proteins [61]. They play significant roles in maintaining the integrity of the host’s intestinal mucosal barrier, supporting the normal function of the host’s immune system, and regulating substance metabolism [62, 63]. Research by Zhai et al. [40] suggests that gut microbes might not be the primary source of circulating microbial DNA, while EVs derived from the GM could have a substantial impact on host health [64, 65]. Variations in flora result in distinct EVs, and dysbiosis could lead to abnormalities in these vesicles, consequently affecting the host differently. Lee and colleagues [66] demonstrated in a preclinical study that transferring fecal microorganisms from elderly mice to young mice increased the likelihood of cognitive deficits, with EVs originating from *Paenalcaligenes hominis* causing hippocampal damage. Furthermore, Wei et al. [67] reported that EVs from the gut microbiota of AD patients activated GSK-3β proteins, induced tau protein phosphorylation, and enhanced the secretion of inflammatory cytokines in the hippocampus.

#### Gut Flora Dysbiosis Impairs Autophagy in Nerve Cells

Autophagy is a protective physiological process that cells undergo in response to stressful conditions, serving as a regulatory mechanism to eliminate unwanted substances and abnormal proteins [37]. Impaired autophagy can lead to inflammatory reactions and cellular dysfunction. Research by Cho et al. [68] showed that dysbiosis of the gut microbiota can hinder autophagy in neuronal cells, with reduced blood butyrate levels in mice with dysbiosis leading to increased CASP3 cleavage protein density and deficits in mitochondrial autophagy. However, supplementation with butyrate was found to improve cognitive deficits in mice. Furthermore, studies by Liu et al. [69] demonstrated that the gut flora and its metabolites play a role in influencing neurological autophagy, impacting the progression of Parkinson’s disease. Interestingly, changes in autophagy in intestinal mucosal cells can also alter the intestinal microecology, resulting in increased intestinal inflammation and systemic immune function alterations [70].

## Anesthesia Interferes with Gut Microecology and Increases the Risk of Postoperative Neurocognitive Impairment

Surgical procedures have a significant impact on the microbiota, with factors such as anesthetic drugs, type and duration of surgery, surgical site, and postoperative medications influencing the diversity of the intestinal flora. Studies by Liu et al. [71] and Wetzel et al. [72] have shown changes in gut microbial composition following surgeries such as sleeve gastrectomy and procedures for treating Crohn’s disease, respectively. These changes were linked to alterations in lipid metabolism and clinical outcomes. Notably, postoperative cognitive deficits may be associated with an increase in proinflammatory bacteria, similar to findings in hosts with cognitive deficits. Furthermore, surgical-induced intestinal mucosal injury can compromise barrier function, allowing toxic metabolites and bacteria to enter the circulation [73, 74].

### Anesthesia Interferes with IntestinalMicroecology

Anesthetic drugs and anesthesia can disrupt the balance of the gut microbiota through direct effects on the microbiota, potentially leading to dysbiosis. Research by Liu et al. [75] suggested that anesthetic drugs have the ability to directly impact the microbiota, potentially causing its death or altering its phenotype. Additionally, the impact of different anesthetic drugs and methods of anesthesia on the microbiota can vary. For instance, a study by Liu et al. [76] compared the effects of sevoflurane, propofol, and a combination of sevoflurane and propofol on postoperative intestinal flora, revealing differences in microbial composition between the groups. Furthermore, the effects of surgical procedures and locations on the intestinal flora can also be significant. In a meta-analysis conducted by Xu et al. [77], the intestinal microbiota of individuals who underwent cholecystectomy exhibited significant differences in 12 species, such as *Escherichia–Shigella*, *Megamonas*, *Prevotella 9,* and *Ruminococcus gnavus*, compared to healthy individuals. In a separate study, Zhang et al. [78] reported that patients who underwent surgery for ventricular septal defect with cardiopulmonary bypass surgery experienced a decrease in the diversity of the intestinal flora during the postoperative period. The abundance of individual bacterial species also underwent significant changes, with a greater abundance of *Enterococci* observed in patients with gastrointestinal dysfunction postoperatively than in those without gastrointestinal dysfunction, while the abundance of *Bifidobacterium* decreased notably. These findings suggest that the impact of surgery on the postoperative intestinal environment varies depending on the surgical site and that interactions between the host and the microbiota lead to alterations in the flora composition.

Opioids can have a notable impact on the GM, leading to disruptions in both microbial and host metabolism. Kolli et al. [79] observed significant increases in the abundances of eight genera, including *Parasutterella excrementihominis*, in the intestinal tracts of mice fed morphine, while the abundance of *Lactobacillus johnsonii* decreased. Wang et al. [80] reported a decrease in the alpha diversity of the intestinal microbiota and a notable increase in potentially pathogenic intestinal flora in mice after morphine administration. The representative genera identified in this study included *Flavobacterium*, *Enterococcus*, *Fusobacterium*, *Sutterella*, and *Clostridioides*.

Surgical stress significantly affects the body’s physiology and metabolism, impacting the host’s stress response to surgery. It also leads to alterations in the body’s microecology, which can greatly influence the recovery and prognosis of postoperative patients.

### Dysregulation of the Microbiota After Anesthetic Surgery Promotes Postoperative Neurocognitive Deficits

**Fig. 1:**
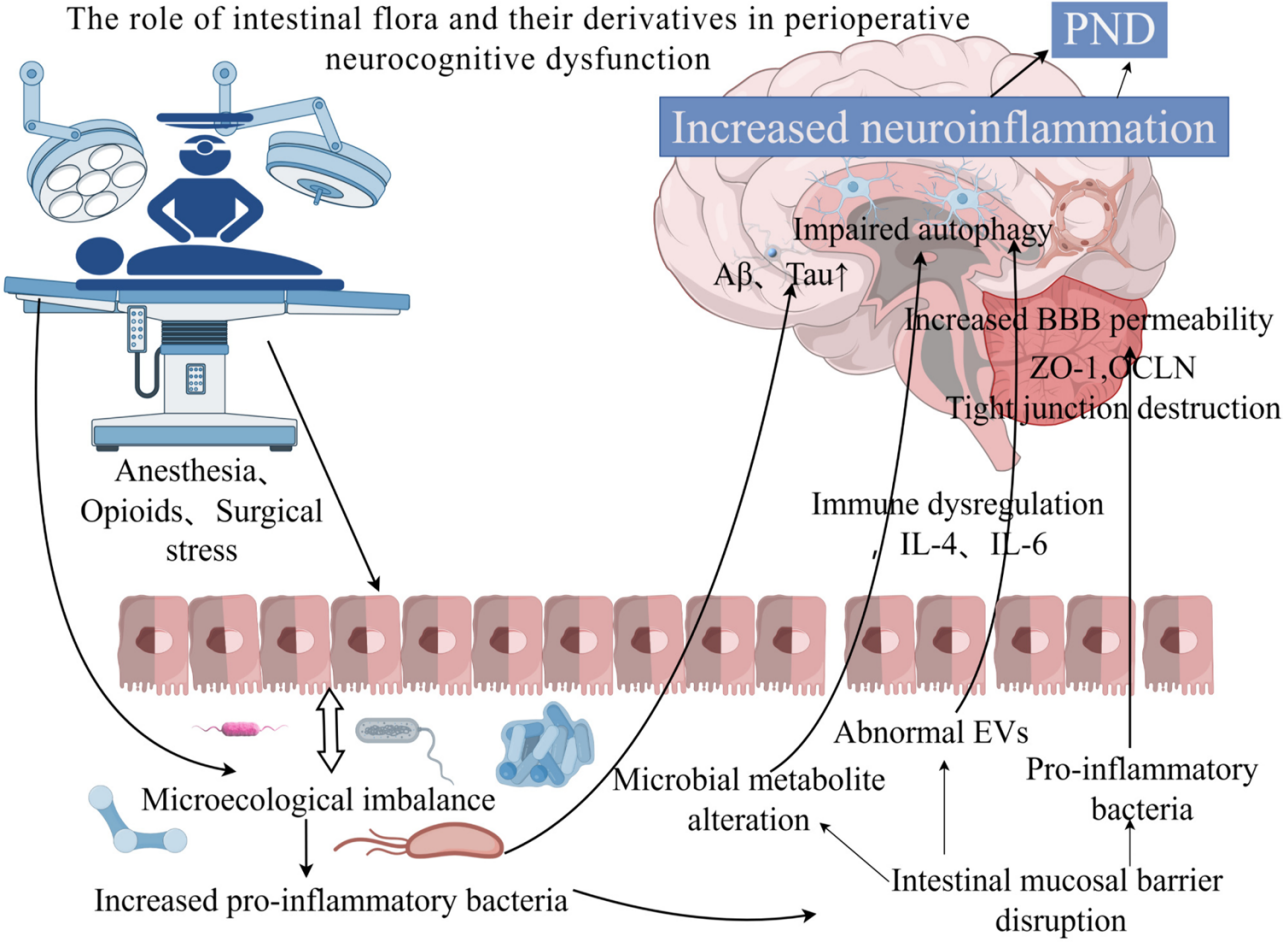
The role of intestinal flora and their derivatives in perioperative neurocognitive dysfunction (by Figdraw). Anesthetic surgical stress and opioid use disrupt the intestinal microecology, leading to an increase in pro-inflammatory flora. This disruption can result in the breakdown of the intestinal mucosal barrier, heightened intestinal permeability, and the circulation of intestinal flora and toxic metabolites, triggering an immune response. This immune response may disrupt the blood‒brain barrier and cause damage to neuronal cells. Additionally, microecological dysregulation can enhance the entry of abnormal cell membrane vesicles into the circulation, promoting neuroinflammatory responses. These combined effects ultimately contribute to the development of neurocognitive deficits

Surgery, while effective in treating many diseases, can also lead to cognitive impairment. The exact mechanisms underlying this phenomenon remain unclear, but potential causes include neuroinflammation and neuronal damage induced by anesthetic drugs during surgery [81], dysregulation of the microecological environment, autophagy deficits in neuronal cells due to surgical and anesthesia-related factors, and immune dysregulation during and after surgery. However, the neurotoxic effects of anesthetic agents remain controversial. Devroe et al. [82] found that sevoflurane anesthesia is an independent risk factor for postoperative delirium in children. Interestingly, Davidson et al. [83] reported that brief exposure to sevoflurane does not affect neurodevelopment and cognitive changes in children. Future studies with larger sample sizes and diverse populations are needed to further investigate this issue. Research has shown that abnormal metabolites in the blood of individuals with dysbiosis of the gut flora can worsen cognitive impairment. For example [84], studies have demonstrated that increased levels of VPA in the blood of mice with postoperative cognitive dysfunction can lead to inflammatory responses in the nervous system and impair learning and memory. Strategies such as exercise and fecal microbial transplantation have been found to reduce blood VPA levels and improve postoperative cognitive function. Furthermore, surgery-induced dysbiosis of the gut flora can affect the ability of EVs to enter the bloodstream [85], with EVs from different flora sources exerting varying effects on the host [86]. Changes in the gut flora composition after surgery have been linked to alterations in the expression levels of proteins involved in maintaining blood‒brain barrier integrity. Treatment with cefazolin has been shown to increase the abundance of bacteria that produce SCFAs in the gut, leading to elevated SCFA levels and partial reversal of postoperative cognitive decline [87] (Fig. 1).

## Personalized Regulation of the Gut Microflora May Be a New Direction for Treating Cognitive Disorders

Research efforts have focused primarily on regulating the intestinal flora and improving diets to prevent and control disease development. However, the selection of specific probiotics or probiotic combinations for various diseases remains a key area requiring further investigation. The evidence suggests the potential for regulating dysbiosis. For instance, Ren et al. [24] demonstrated in a preclinical trial that modifying the intestinal flora through dietary changes led to positive outcomes, potentially due to repairing intestinal flora disorders, reducing neuroinflammation, and enhancing nerve function. Similarly, Bosch et al. [88] reported that the administration of GV-971 to mice altered the intestinal flora, reduced Aβ protein deposition, alleviated neuroinflammation, and improved neurocognitive deficits by targeting the microbiota–microglia–amyloid axis. A study from Korea [89] confirmed the role of probiotics in liver function protection, showing that consuming beverages with *L. holzapfelii* preparations restored the intestinal flora structure and decreased serum transaminase levels. Notably, the effectiveness of a single probiotic species may be limited, and the same flora may not have consistent effects across different organs and systems. For example, a study by Johnstone and colleagues [90] revealed no significant difference in therapeutic efficacy between *Lactobacillus rhamnosus GG* and a placebo in improving respiratory-associated pneumonia. *Lactobacillus rhamnosus GG* showed no significant difference in therapeutic effect compared to the placebo*.* Further research studies are required to better understand the role of gut flora in various groups and diseases.

## Summary

In conclusion, dysregulation of the gut microbiota induces PND. Direct interactions between the microbiota and the host, where microbial metabolites stimulate the host to produce an immune-inflammatory response, may be the key mechanism in this induction. This process involves the disruption of gut mucosal barrier function and increased permeability of the blood‒brain barrier, allowing toxic metabolites and abnormal extracellular vesicles to enter the body’s circulation. Surgery and anesthesia can disrupt the intestinal microecology, leading to an ecological imbalance that may initiate PND. Therefore, implementing appropriate preoperative and postoperative interventions to minimize damage to the microbiota ecosystem and repair microecological disturbances could be promising therapeutic strategies. Further clinical and experimental studies are necessary to provide new evidence for standardizing the protection and adjustment of the intestinal flora, as well as identifying specific therapeutic targets for the standardized management of PND.

## Data Availability

No datasets were generated or analyzed during the current study.
